# Secretory Cavity Development and Epidermal Exudation Pathways in Fruits of *Ruta graveolens* L. (Rutaceae)

**DOI:** 10.3390/plants15132084

**Published:** 2026-07-03

**Authors:** Silvia Rodrigues Machado, Aline Rodrigues de Almeida, Karla Bianca de Deus Bento, Sabrina Lemes Dias, Tatiane Maria Rodrigues

**Affiliations:** 1 Electron Microscopy Center, Institute of Biosciences of Botucatu, São Paulo State University (UNESP), Botucatu 18618-970, SP, Brazil; 2 Interunit Postgraduate Program in Plant Biology, Institute of Biosciences of Botucatu and Rio Claro, São Paulo State University (UNESP), Botucatu 18618-970, SP, Brazil; aline.r.almeida@unesp.br; 3 Department of Biodiversity and Biostatistics, Institute of Biosciences of Botucatu, São Paulo State University (UNESP), Botucatu 18618-970, SP, Brazil; kb.bento@unesp.br (K.B.d.D.B.); sabrina.lemes@unesp.br (S.L.D.); tatiane.rodrigues@unesp.br (T.M.R.)

**Keywords:** oil glands, secretion dynamics, surface exudation

## Abstract

*Ruta graveolens* L. fruits are densely covered with translucent dots corresponding to secretory cavities that accumulate bioactive metabolites, primarily essential oils. Immature fruits present an aromatic surface exudate, indicating the active release of secretory products despite the internal location of the cavities. This study investigated the origin, development, ultrastructure, and secretion-release mechanisms of fruit secretory cavities using light, scanning, and transmission electron microscopy. Secretory cavities originated from clusters of ground meristem cells associated with phloem strands. Lumen formation began with the collapse of central cells, while surrounding cells differentiated into a metabolically active secretory epithelium rich in polymorphic plastids, smooth endoplasmic reticulum, mitochondria, vesicles, and lipid bodies. Mature cavities consisted of a multilayered epithelium surrounding a large lumen and enclosed by a parenchymatous sheath. Progressive lysis of inner epithelial cells contributed to lumen expansion and secretion accumulation. As cavities enlarged, they became positioned immediately beneath the epidermis, whose cells became compressed and flattened. Secretion was released through the rupture of glandular and epidermal cells and through stomata located in epicarp depressions. Ultrastructural evidence indicates the combined operation of eccrine, granulocrine, and holocrine secretion mechanisms. Pectin–cellulosic wall thickenings likely function as apoplastic barriers, directing secretion toward the lumen and protecting adjacent tissues.

## 1. Introduction

Specialized metabolites produced by secretory structures in terrestrial plants play a central role in plant defense, contributing to both constitutive and inducible responses against herbivores and pathogens. The ability to compartmentalize specialized metabolites and defense-related proteins within secretory structures represents a key evolutionary innovation, profoundly influencing the dynamics of plant–herbivore and plant–pathogen interactions [1].

Secretory cavities are among the most prominent internal secretory structures in plants. They are characterized by a layer of specialized epithelial cells (secretory epithelium) surrounding an intercellular space (lumen), where secretions accumulate on the surface [2,3]. Unlike external secretory structures, the secretion is retained within the lumen rather than being directly released to the plant. A secretory cavity may develop through schizogeny, lysigeny, or schizolysigeny, depending on the relative contribution of cell separation and cell lysis to lumen formation. Secretion release generally occurs through merocrine mechanisms, without cell disruption, although holocrine secretion, involving the disintegration of secretory cells, has also been reported in several plant secretory systems [3].

Secretory cavities have been reported in diverse families such as Asteraceae, Hypericaceae, Leguminosae, Myrtaceae, and Rutaceae [4]. In Rutaceae, these structures, called oil glands, are the principal sites of essential oil synthesis and storage. Essential oils produced within the secretory cavities play an important role in plant defense, owing to the antibacterial, antifungal, and insecticidal activities of their constituents [5,6]. Essential oils from several Rutaceae, with a focus on *Citrus* species, are also widely used as additives in the food, pharmaceutical and cosmetic industries [7,8].

*Ruta graveolens* L., commonly known as rue or garden rue, is an aromatic shrub widely distributed worldwide and cultivated as both a medicinal and ornamental species [9]. The species is a rich source of secondary metabolites, including coumarins, alkaloids, volatile oils, flavonoids, and phenolic acids, which underpin diverse pharmacological properties [10]. Beyond its medicinal applications, *R. graveolens* also holds cultural significance in religious practices [11]. Pharmacological studies have demonstrated a broad spectrum of biological activities, including anthelmintic, febrifuge, emmenagogue, and abortifacient effects [10,12,13,14]. Notably, the species is considered a promising source of furanocoumarins, compounds widely used in dermatology for the symptomatic treatment of severe vitiligo, psoriasis, and mycosis fungoides [15]. These metabolites accumulate predominantly in the fruits, where their concentrations can be five to ten times higher than those found in leaves, stems, and roots, contributing to fruit toxicity and explaining their contraindication during pregnancy [16].

The fruits of *R. graveolens* are coarse-textured, lobed dehiscent capsules, typically consisting of four to five mericarps densely covered with pellucid glands appearing as translucent dots [17]. Studies in Rutaceae have demonstrated that such translucent dots are secretory cavities [18]. A hyaline fluid exudate was observed on the surface of immature fruits in regions corresponding to the translucent dots (personal observations). This exudate emits the characteristic rue odor, which becomes markedly more intense during the hottest hours of the day, suggesting a close association with glandular activity and essential oil secretion. The presence of a surface exudate thus establishes a clear conceptual tension: if secretion is confined to an internal compartment, how does it reach the organ’s surface? This apparent inconsistency raises fundamental questions regarding both the origin of the exudate and the mechanisms governing its externalization, highlighting the need for a more integrative structural and developmental perspective.

In this context, the present study examines fruits of *R. graveolens* across different developmental stages, aiming to elucidate the origin, cytological differentiation and functional dynamics of secretory cavities, as well as the mechanisms underlying surface exudation.

## 2. Results

**Figure 1:**
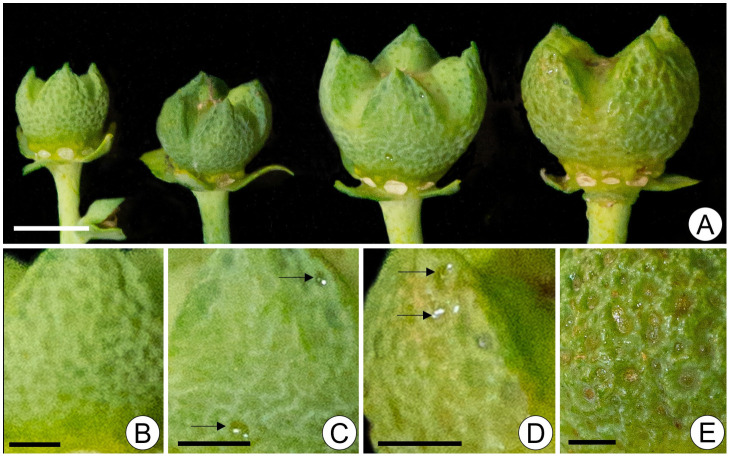
(**A**–**E**) Fruits of *Ruta graveolens* at different developmental stages. The arrows indicate exudate accumulated within depressions on the fruit surface in (**C**,**D**). In (**E**), exudate is spread across the fruit surface. Scale bars: (**A**,**D**) = 0.5 cm; (**B**) = 0.1 cm; (**C**,**E**) = 0.3 cm.

In the collection area, fruits of *R. graveolens* were produced almost continuously throughout the year, with a marked peak occurring from October to March. Fruits at different developmental stages were often observed on the same branch. Translucent dots were evident on the fruit surface and became progressively more conspicuous during development (Figure 1A–D). The exudate initially accumulated within the depressions between the protuberances (Figure 1C,D) and later spread across the fruit surface.

### 2.1. Micromorphology of Fruit Surface

**Figure 2:**
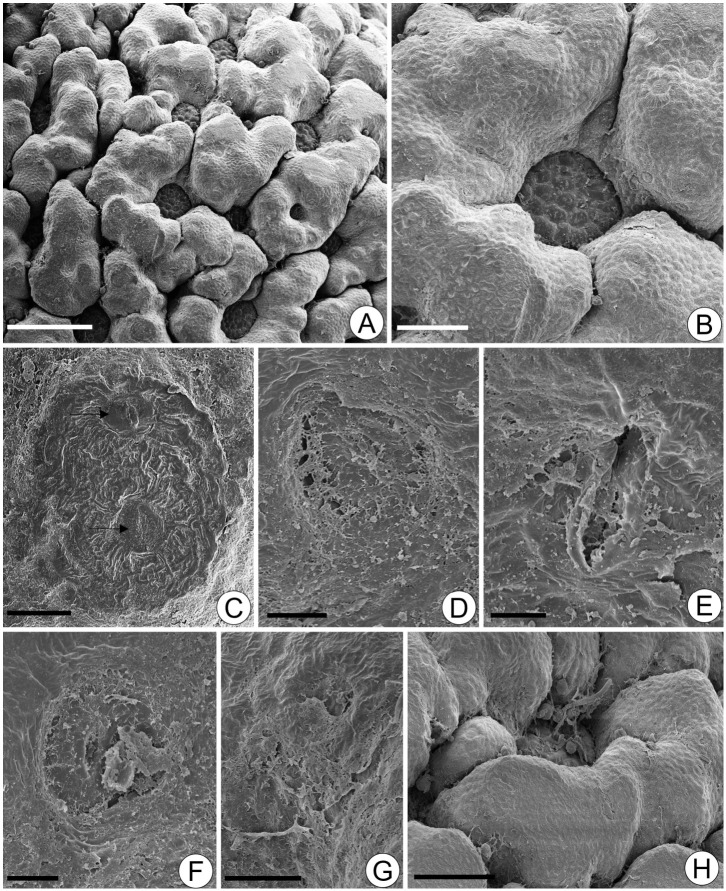
(**A**–**H**) Scanning electron micrographs of the surface of *Ruta graveolens* fruits. (**A**) General view showing juxtaposed protuberances delimiting depression areas. (**B**) Details of previous figure showing depression area encircled by protuberances. (**C**) Depression area covered by striated cuticle and containing stomata (arrows). (**D**) Depression area with gaps on its surface. (**E**) Fissure observed in depression area. (**F**) Residual exudate covering depression area. (**G**) Fruit surface covered by residual exudate. (**H**) Pollen grains and fungi hyphae inside depression. Scale bars: (**A**) = 250 µm; (**B**) = 70 µm; (**C**,**G**) = 20 µm; (**D**–**F**) = 5 µm; (**H**) = 100 µm.

Scanning electron microscopy revealed that the fruit surface was characterized by closely juxtaposed protuberances delimiting depressions (Figure 2A,B), which were lined by a distinctly striated cuticle and bore stomata (Figure 2C). These depressions also exhibited gaps (Figure 2D) or fissures (Figure 2E), which were frequently obstructed by residual exudate (Figure 2F). Residual secretion was additionally observed across the entire fruit surface (Figure 2G). Pollen grains, fungal spores, and hyphae were commonly observed within the depressions (Figure 2H).

### 2.2. Ontogenesis and Development of Secretory Cavities

**Figure 3:**
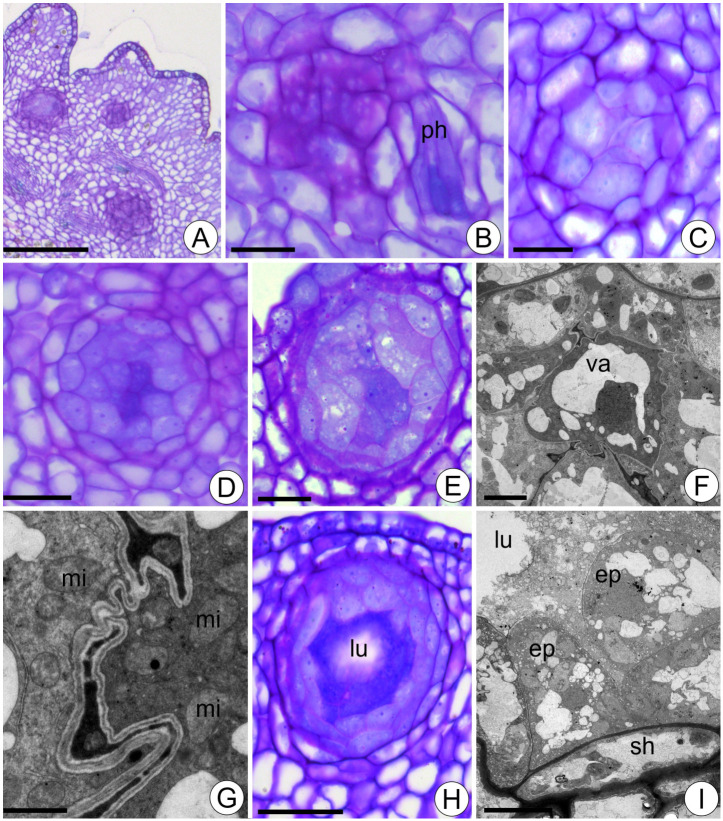
(**A**–**I**) Photomicrographs of longitudinal sections stained with toluidine blue of *Ruta graveolens* pericarp (**A**–**E**,**H**) and transmission electron micrographs (**F**,**G**,**I**) of developing secretory cavities. (**A**) Secretory cavities at different developmental stages distributed throughout the pericarp. (**B**) Primordial cells with thick walls and dense cytoplasm. Observe the strand of phloem near the primordial cells. (**C**) A cluster of primordial cells with enlarged central cells. (**D**) A cluster of primordial cells with central cells more intensely stained. (**E**) Clustered primordial cells with voluminous nucleus, small vacuoles, and dense cytoplasm. (**F**) Central cells in the cluster with sinuous cell walls, electron-dense cytoplasm, and vacuole. (**G**) Details of the central cells of the cluster showing very sinuous walls and dense cytoplasm with abundant mitochondria. (**H**) Developing lumen in the central region of the cluster. (**I**) Pyramidal cells arranging around the developing lumen. ep: epithelial cell; lu: lumen; mi: mitochondria; ph: phloem; sh: parenchyma sheath; va: vacuole. Scale bars: (**A**) = 200 µm; (**B**,**D**,**E**) = 25 µm; (**C**,**I**) = 20 µm; (**F**) = 10 µm; (**G**) = 2.5 µm; (**H**) = 50 µm.

In young fruits, secretory cavities at different developmental stages were distributed throughout the pericarp (Figure 3A). The initial cells of the secretory cavities were readily distinguished from neighboring cells by their pyramidal shape, thick cell walls, and dense cytoplasmic content (Figure 3B). Strands of phloem were closely associated with these primordial cells (Figure 3B). At a subsequent developmental stage, the primordial cells became arranged in a cluster, with enlarged central cells clearly evident (Figure 3C). The primordial cells became more numerous, and the central cells of the cluster stained more intensely with toluidine blue (Figure 3D). The clustered cells were voluminous and exhibited a spherical nucleus with a prominent nucleolus, small vacuoles, and dense cytoplasm (Figure 3E). These cells divided in several planes, giving rise to immediate derivative cells of different sizes and shapes (Figure 3E). Ultrastructurally, the central cells of the cluster were characterized by sinuous cell walls, electron-dense cytoplasm distributed along the cell periphery, a large vacuole traversed by cytoplasmic strands (Figure 3F), ovoid plastids lacking thylakoids, and numerous mitochondria (Figure 3G). Subsequently, the central cells collapsed, giving rise to a small central space (Figure 3H,I) from which the cavity lumen originated. The cells adjacent to the developing lumen became pyramidal in shape, exhibiting distinct basal and apical poles (Figure 3I). These cells were characterized by the presence of numerous small vacuoles and oil droplets distributed throughout the cytoplasm and within the vacuoles (Figure 3I).

**Figure 4:**
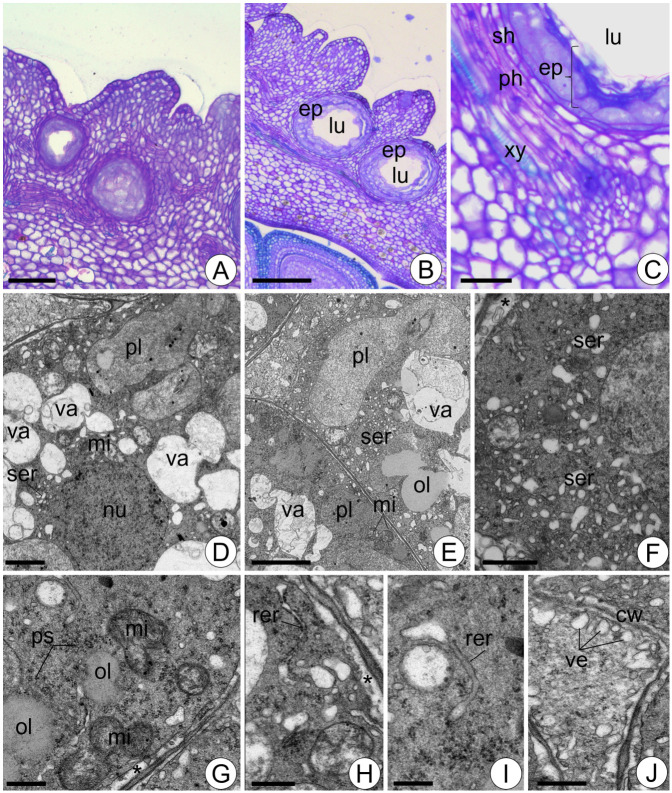
(**A**–**J**) Photomicrographs of longitudinal sections stained with toluidine blue of *Ruta graveolens* pericarp (**A**–**C**) and transmission electron micrographs (**D**–**J**) of secretory cavities. (**A**) Secretory cavities in different developmental stages in the pericarp. (**B**) Secretory cavities in the outer portion of the pericarp with multiseriated epithelium and lumen. (**C**) The secretory cavity associated with the vascular tissues. (**D**) Epithelial cells with very thin walls, a nucleus, and an abundant cytoplasm with polymorphic plastids, a smooth endoplasmic reticulum, mitochondria and vacuoles. (**E**) Portions of epithelial cells with large and polymorphic plastids, mitochondria, an abundant smooth endoplasmic reticulum, vacuoles and oil bodies. (**F**) Details of an epithelial cell with an abundant smooth endoplasmic reticulum with dilated cisterns and derived vesicles. Observe paramural bodies in the periplasmic space. (**G**) An epithelial cell with mitochondria (mi), polysomes and oil drops in the cytoplasm. (**H**,**I**) A rough endoplasmic reticulum with bulbous ends. (**J**) Abundant vesicles juxtaposed to the plasma membrane. cw: cell wall; ep: epithelium; lu: lumen; mi: mitochondria; nu: nucleus; ol: oil; ph: phloem; pl: plastid; ps: polysomes; rer: rough endoplasmic reticulum; ser: smooth endoplasmic reticulum; va: vacuole; ve: vesicles; xy: xylem. The * indicate dilated periplasmic space. Scale bars: (**A**) = 100 µm; (**B**) = 200 µm; (**C**) = 30 µm; (**D**,**F**) = 2 µm; (**E**) = 5 µm; (**G**,**H**,**J**) = 1 µm; (**I**) = 0.5 µm.

As cluster differentiation progressed, the central space enlarged (Figure 4A). Simultaneously, the cavities increased in size and appeared to shift toward the outer portion of the mesocarp (Figure 4B), while remaining closely associated with the vascular tissues (Figure 4C). Differentiated secretory cavities consisted of an epithelium composed of two to three layers of secretory cells surrounding a large lumen (Figure 4B). Ultrastructurally, the epithelial cells exhibited very thin walls, a spherical nucleus (Figure 4D), and abundant cytoplasm (Figure 4E–J) rich in polymorphic plastids, a dilated smooth endoplasmic reticulum (Figure 4D–F), globular mitochondria, and polysomes (Figure 4G). Rough endoplasmic reticulum was scarce and frequently exhibited bulbous ends (Figure 4H,I). Vesicles derived from the endoplasmic reticulum were concentrated in the peripheral cytoplasm and were frequently observed to be juxtaposed to the plasma membrane (Figure 4J). Small vacuoles containing flocculent material and cell debris were also present (Figure 4D,E). Lipid bodies were distributed throughout the cytoplasm and within the vacuoles (Figure 4E,G). Paramural bodies were observed in the periplasmic space (Figure 4H).

**Figure 5:**
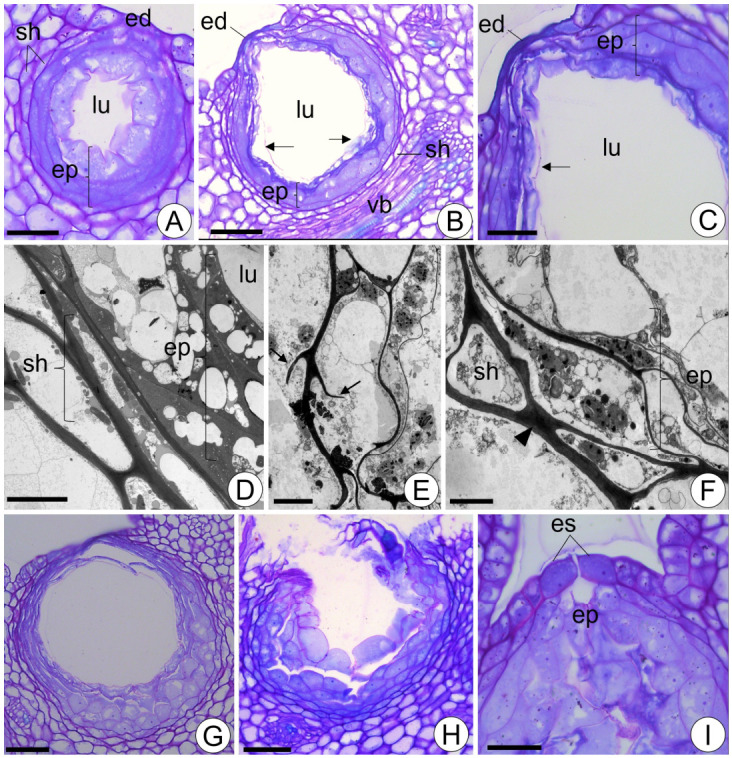
(**A**–**I**) Photomicrographs of longitudinal sections stained with toluidine blue of *Ruta graveolens* pericarp (**A**–**C**,**G**–**I**) and transmission electron micrographs (**D**–**F**) of secretory cavities. (**A**) A secretory cavity with an established lumen and multiseriate epithelium located immediately beneath the epidermis. (**B**) A secretory cavity with flattened inner epithelial cells. Observe the contact between the sheath of parenchyma cells and the vascular tissues. (**C**) Details of the previous figure showing inner epithelial cells with lysis signals and flattened epidermal cells. (**D**) Flattened epithelial cells with very thin walls and densely packed cytoplasm. Observe the parenchyma sheath subjacent to the epithelium. (**E**) Epithelial cells with peg-like projections composed of wall material. (**F**) Parenchyma sheath cells with bands of polysaccharide thickenings (arrowhead). (**G**) A secretory cavity showing the detachment of the epithelial cells. (**H**) A secretory cavity with a ruptured epithelium. Observe also the rupture of the epidermis. (**I**) Stomata on the epidermis just above the secretory cavity. ed: epidermis; ep: epithelium; es: stomata; lu: lumen; sh: parenchyma sheath; vb: vascular bundle. The arrows indicate cell debris in the lumen. Scale bars: (**A**,**C**,**I**) = 25 µm; (**B**,**G**,**H**) = 50 µm; (**D**,**E**) = 10 µm; (**F**) = 5 µm.

As the differentiation process progressed, the inner epithelial cells became more voluminous and exhibited irregular shapes and outlines (Figure 5A). Their nuclei were spherical and conspicuous, with prominent nucleoli (Figure 5A). The subjacent epithelial layers became flattened and more densely stained (Figure 5A). At a later stage, the inner epithelial cells underwent lysis, and cell debris was released into the lumen (Figure 5B,C). Externally, the cavities were enclosed by a sheath composed of one to two layers of parenchyma cells (Figure 5A,B). This parenchymatous sheath remained in contact with the phloem of the collateral vascular bundles (Figure 5B). Mature secretory cavities became concentrated immediately beneath the epidermis (Figure 5A–C). At the distal pole of the secretory cavities, the epithelial cells became flattened and were displaced outward, causing the epidermis to assume a convex appearance (Figure 5B,C). Ultrastructurally, the flattened epithelial cells exhibited very thin walls and densely packed cytoplasm, in which organelles were difficult to distinguish (Figure 5D). Some epithelial cells exhibited peg-like projections composed of wall material (Figure 5E). The anticlinal walls of the sheath cells were relatively thicker and exhibited bands of polysaccharide thickenings (Figure 5F).

Finally, the epidermal cells also became flattened (Figure 5G) and ruptured (Figure 5H), leading to the release of the secretion onto the fruit surface. Secretion could also be released through stomata located in depressions of the pericarp (Figure 5I).

## 3. Discussion

Our study demonstrated that secretory cavities of *Ruta graveolens* are established early during fruit development, whereas cavity enlargement and oil accumulation intensify during later stages of fruit expansion. The relationship between oil gland and fruit development was observed in several *Citrus* species [19,20].

Our findings indicate that secretory cavities originate from subepidermal ground-meristem initials through a coordinated developmental program. Their ontogeny begins with cell enlargement, followed by coordinated cell divisions and progressive cellular differentiation accompanied by the darkening of the cytoplasm and subsequent collapse of the central cells, which leads to the formation of the cavity lumen, whereas further lumen expansion results from the lysis of inner epithelial cells, characterizing a lysigenous mode of development. A similar pattern of formation of secretory cavities was described in the leaves of *Dictamnus dasycarpus* (Rutaceae) [21]. Our observations help clarify long-standing divergences concerning both the origin of the cavities and the mechanisms underlying lumen formation in secretory cavities of Rutaceae species, which have been interpreted as lysigenous, schizogenous or schizolysigenous [22].

During cavity maturation, the innermost epithelial cells undergo progressive lysis and are shed into the lumen, where they eventually degenerate. This lysigenous process not only contributes cellular content to the secretion but also promotes lumen enlargement. Furthermore, secretion release in *Ruta graveolens* fruits involves a holocrine mechanism, as previously described for resin canals of Anacardiaceae and Calophyllaceae [23,24,25]. The progressive degeneration of epithelial cells observed during cavity development is consistent with the occurrence of programmed cell death (PCD), a process that has been recognized as a key mechanism in the formation and expansion of secretory cavities in *Citrus* species [21,26,27,28].

During fruit development, the secretory cavities become progressively enlarged, assume an ovoid shape, and become positioned immediately beneath the epidermis. At advanced developmental stages, the sheath cells and epithelial cells located in the distal region of the cavity adjacent to the epidermis undergo progressive collapse. Simultaneously, continued accumulation of secretion within the lumen likely generates mechanical pressure against the overlying tissues. All these events culminate in epidermal and epithelial cell ruptures, releasing the cavity contents onto the fruit surface, a process consistent with holocrine secretion [2]. Eventually, secretion discharge may also occur through stomatal openings, thereby establishing direct pathways for secretion extrusion to the fruit surface. A comparable mechanism of epidermal extrusion associated with epidermal disruption has also been described for resin-secreting cavities in the androecium of *Clusia* (Clusiaceae) [29].

The physiological implications of this epidermal extrusion mechanism may be interpreted from complementary perspectives. On one hand, the rupture of the epidermis compromises tissue continuity and temporarily weakens the physical barrier of the fruit surface, potentially increasing susceptibility to pathogen entry and other environmental stresses. On the other hand, the secretion released onto the surface is rich in bioactive lipophilic compounds that may compensate for this transient structural disruption by forming a chemically protective layer. Thus, secretion release through epidermal rupture appears to represent a functional trade-off between temporary loss of physical integrity and the establishment of an effective chemical defense at the fruit surface [30].

The ultrastructural organization of the epithelial cells in *Ruta graveolens* is consistent with that of highly metabolically active cells described in other secretory systems [3]. These cells exhibit dense cytoplasm, large nuclei, polymorphic plastids containing lipid globules, abundant smooth (SER) and rough endoplasmic reticulum (RER), scattered lipid bodies, and relatively few dictyosomes, features commonly associated with oil-secreting glands in diverse taxa [31,32,33,34,35,36]. The extensive development of the SER highlights its central role in lipid synthesis and transport, enabling efficient production and secretion of lipophilic compounds [37]. Because these compounds can diffuse across the plasma membrane and cell wall, part of the secretion process likely occurs through an eccrine mechanism [2]. The prominent RER and associated vesicles further indicate intense secretory activity. In eukaryotic cells, the RER is directly involved in protein synthesis and intracellular trafficking, while vesicles derived from the endoplasmic reticulum mediate the transport of proteins and lipids through the secretory pathway [37,38]. In *R. graveolens*, the presence of ER-derived vesicles adjacent to or fused with the plasma membrane suggests that part of the secretion is released by exocytosis into the periplasmic space, characterizing a granulocrine mechanism [2]. After traversing the cell wall, the secretion accumulates within the lumen, where its progressive buildup likely generates the mechanical pressure associated with cell rupture during secretion release [39]. The occurrence of RER and numerous polyribosomes also suggests active synthesis of hydrolytic enzymes, mainly pectinases and cellulases, involved in cell wall degradation during lumen expansion and tissue collapse [40].

The pectin–cellulosic thickenings observed in the anticlinal and inner periclinal walls of epithelial cells, as well as in the sheath surrounding the secretory cavities of *Ruta graveolens*, are similar to those described for the secretory canals of *Protium ovatum* (Burseraceae) [41]. These wall modifications likely function as apoplastic barriers that direct secretion flow toward the lumen while preventing the diffusion of metabolites into adjacent tissues. Such compartmentalization may be particularly important in protecting surrounding cells from the toxic effects of secreted compounds, especially furanocoumarins, which are known for their high biological activity and toxicity in the fruits of rue [16].

## 4. Materials and Methods

### 4.1. Plant Material

Immature fruits at different developmental stages, ranging from <10 mm to ~70 mm in length, were collected from five individuals of *Ruta graveolens* cultivated in a particular area located in the Palos Verdes Garden, municipality of Botucatu (22°53′09″ S 48°26′42″ W), São Paulo State, Brazil.

After collection, the fruits were immediately dissected, and small fragments were promptly immersed in appropriate fixative solutions for anatomical and ultrastructural studies.

Vouchers were deposited in the BOTU Herbarium “Irina Delanova Gemtchújnicov” of the Institute of Biosciences, UNESP, Botucatu, Brazil.

### 4.2. Anatomical Studies

For light microscopy (LM), fruit samples were fixed in 2.5% glutaraldehyde in 0.05 M sodium phosphate buffer for 24 h [42]. The material was dehydrated through a graded ethanol series and embedded in (2-hydroxyethyl) methacrylate historesin (Leica Microsystems, Nussloch, Germany) following standard protocols. Cross and longitudinal sections (5 μm thick) were obtained using a semiautomatic microtome (Leica RM2245, Leica Biosystems, Nussloch, Germany), stained with 0.05% Toluidine Blue at pH 4.2 [43], and mounted in synthetic resin (Entellan, Merck KGaA, Darmstadt, Germany) on permanent slides. Observations and image acquisition were carried out using a light microscope (Leica DMR, Leica Microsystems, Wetzlar, Germany) equipped with a digital camera (Leica DFC 500, Leica Microsystems, Wetzlar, Germany).

### 4.3. Scanning Electron Microscopy (SEM)

Fruit surface analyses were performed using scanning electron microscopy (SEM, Thermo Fisher Scientific, Waltham, MA, USA). Samples were fixed in 2.5% glutaraldehyde in 0.1 M phosphate buffer (pH 7.2), dehydrated in graded ethanol, and critical-point dried (Bal-Tec CPD 030, Balzers, Liechtenstein). The dried material was mounted on aluminum stubs, sputter-coated with gold (10 nm) (Bal-Tec SCD 050, Balzers, Liechtenstein), and examined using a SEM (FEG, Thermo Fisher Scientific, Waltham, MA, USA) at 20 kV.

### 4.4. Transmission Electron Microscopy (TEM)

For ultrastructural analysis of the glands, samples were fixed in a solution containing 2% (*v*/*v*) glutaraldehyde and 4% (*w*/*v*) paraformaldehyde in 50 mM sodium phosphate buffer (pH 7.4) overnight at 4 °C. The material was post-fixed in 1% (*w*/*v*) osmium tetroxide in the same buffer, dehydrated in a graded ethanol series, and embedded in Araldite resin (Araldite 502, Electron Microscopy Sciences, Hatfield, PA, USA). Ultrathin sections were contrasted with uranyl acetate (4% *w*/*v*) and lead citrate [44] and examined using a transmission electron microscope (Tecnai Spirit, FEI Company, Brno, Czech Republic) operating at 80 kV.

## 5. Conclusions

Our study reveals that the secretory cavities of *Ruta graveolens* fruits constitute highly dynamic structures whose development, secretion synthesis, and release are tightly integrated throughout fruit ontogeny. Cavity formation is predominantly lysigenous, while secretion release results from the coordinated action of eccrine, granulocrine, and holocrine mechanisms. The progressive accumulation of secretion ultimately leads to tissue rupture and extrusion of bioactive compounds onto the fruit surface, suggesting a specialized strategy for chemical defense. By elucidating the developmental origin, secretory mechanisms, and functional organization of these cavities, this work resolves longstanding questions regarding secretory cavity ontogeny in Rutaceae and provides a framework for understanding the ecological and evolutionary significance of internal secretory systems in aromatic plants.

## Data Availability

The original contributions presented in this study are included in the article. The raw images are available from the corresponding author upon reasonable request.
